# Intraoperative Radiation Therapy (IORT) for Breast Cancer: The Final Analysis of a Prospective Cohort of 1828 Cases

**DOI:** 10.1245/s10434-025-17546-9

**Published:** 2025-05-31

**Authors:** Melvin J. Silverstein, Brian Kim, Kevin Lin, Shane Lloyd, Lincoln Snyder, Sadia Khan, Deena Hossino, Peter Chen

**Affiliations:** 1 Department of Surgery, Hoag Memorial Hospital Presbyterian, Newport Beach, CA USA; 2 Department of Surgery, Keck School of Medicine, University of Southern California, Los Angeles, CA USA; 3 Department of Radiation Oncology, Hoag Memorial Hospital Presbyterian, Newport Beach, CA USA

## Abstract

**Background:**

Intraoperative radiation therapy (IORT) delivers the full course of radiation therapy during the initial surgical excision. In the early 2000s, IORT was a promising method of breast cancer treatment de-escalation, offering many advantages. This technique should have succeeded in the USA, but several factors hindered its widespread adoption. We report the results of our 15-year IORT registry trial and our reasons for halting the trial.

**Patients and Methods:**

Patients with early-stage breast cancer were entered into an institutional review board (IRB) approved registry. They were analyzed by intention to treat and by various subgroups, including those who received local treatment to the area of the primary tumor only versus those who received additional whole breast treatment.

**Results:**

A total of 1785 patients with 1828 early-stage breast cancers entered the registry. With a median follow-up of 85 months, the local recurrence rate (LRR) at 5-years for the entire cohort was 4.44%. Among 1527 who received local treatment only, the LRR at 5-years was 5.09% compared with 1.13% for 301 patients who received additional whole breast treatment (*p* = 0.001). For patients aged ≥ 65 with luminal A tumors ≤ 20mm, the 5-year LRR dropped to 2.32%.

**Conclusions:**

IORT is extremely convenient for the patient and offers many advantages when compared with other methods of partial or whole breast treatment. Overall, the LRR for IORT is 4–5 times higher than many competing forms of whole or accelerated partial breast irradiation but still relatively low. A more select choice of patients for IORT can lower the LRR. Following the decision by ASTRO in 2024 not to recommend IORT, and considering a number of other factors, our group made the decision to discontinue our IORT program.

During the first two decades of this century, intraoperative radiation therapy (IORT) emerged as a valuable tool in the de-escalation of treatment for early-stage breast cancer. Two prospective randomized IORT trials, TARGIT-A ^1–3^ and ELIOT^4–6^ showed promising results, with acceptable local recurrence rates. Despite this, IORT never gained a strong foothold in the USA. While its use expanded in Europe, IORT has nearly disappeared in this country. This decline resulted from a combination of factors, including higher local recurrence rates for IORT compared with external beam radiation therapy, low physician and hospital reimbursement, and the development of competing hypofractionated radiation techniques.

The failure of IORT to gain widespread use is surprising given the trend to de-escalate therapy in breast cancer treatment over the past several decades. Since the mid-1970s, there has been steady movement toward de-escalation, shifting away from radical approaches in favor of more conservative strategies. Mastectomies have become less common, excision with radiation therapy replaced more extensive surgeries, oncoplastic breast cancer surgery has become popular, axillary dissections have been minimized in favor of sentinel lymph node biopsies, and hypofractionated radiation shortened treatment duration. Recently there has been some support for excision alone, without sentinel lymph node biopsy or radiation therapy, in highly selected low-risk patients.^7–9^

IORT was an innovative tool that fit naturally into the de-escalation trend. By delivering the full course of radiation therapy in a single dose during initial cancer surgery, it eliminated the need for multiple visits to a radiation center, minimized potential damage to surrounding organs, and made breast conservation a viable option for women who lived far from treatment facilities or had demanding schedules. However, several factors hindered its widespread adoption. Initially, the lack of long-term data made radiation oncologists hesitant to embrace IORT, particularly when alternative balloon brachytherapy techniques such as MammoSite^®^ were already established.^10^ Reimbursement was initially poor and never significantly improved. Finally, controversy surrounding the data and statistical interpretations from the TARGIT-A trial cast doubt on IORT’s efficacy, ultimately limiting its widespread acceptance.^11–13^

A major blow to IORT’s acceptability came with the 2024 update of the American Society of Radiation Oncology’s (ASTRO) Practice Guidelines for Partial Breast Irradiation.^14^ ASTRO concluded that recurrence rates were too high for IORT when compared with other forms of radiation therapy and stated that it should only be performed within a clinical trial or multicenter registry. The International Society of Intraoperative Radiation Therapy Working Group strongly opposed this recommendation^15^, advocating for the continuation of ASTRO’s 2017 “conditional recommendation”.^16^ This objection had little impact on IORT’s decline in the USA and ASTRO did not change its position.

In 2009, our team of breast surgical and radiation oncologists carefully evaluated the early data and available equipment. In 2010, we launched an institutional review board (IRB)-approved IORT registry trial. Over the years, we met regularly to assess our results and the published results of others. We continued the program yearly, publishing multiple studies.^17–19^ However, in 2024, following publication of the updated 2024 ASTRO guidelines,^14^ we made the difficult decision to discontinue our trial. In this paper, we present the final report on our prospective cohort and we discuss why we decided to discontinue our trial.

## Patients and Methods

Patients with early-stage breast cancer with a diagnosis of invasive ductal, invasive lobular, or ductal carcinoma in situ (DCIS), or a combination of these, were brought to the operating room for surgical excision of their primary tumor and treatment with IORT at Hoag Memorial Hospital Presbyterian, Newport Beach, California. They were accrued to an institutional review board approved tumor registry and met the guidelines of their responsible governmental agency. Detailed informed consent was obtained from every patient. The trial was designed in 2010 and did not include collection of data on race or ethnicity. All patients were women.

### Protocol Requirements

**Fig. 1:**
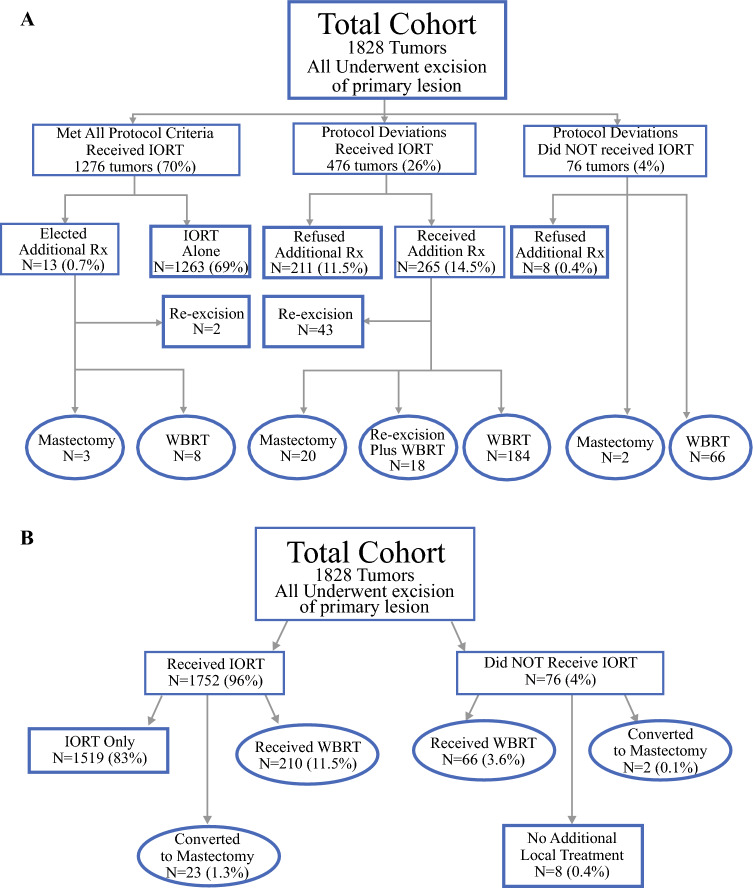
**A** Flow chart. 1828 breast cancers underwent surgical excision, and initially were divided into three groups: (1). 1276 tumors that met all IORT protocol criteria, (2). 476 tumors that deviated from 1 or more IORT protocol criteria on final histopathology but were given IORT, and (3). 76 tumors that deviated from IORT protocol criteria discovered in the operating room and were not given IORT; those that ended up in bold rectangular boxes with bold letters received partial breast treatment as their final breast treatment, and those that ended up in bold ovals with bold letters received whole breast treatment as their final breast treatment; **B** simplified version of **A** multiple groups have been combined to yield four bold ovals with bold letters with a total 301 patients who received whole breast treatment; there are two bold rectangular boxes with bold letters with a total 1527 patients who received partial breast treatment. Additional Rx = additional treatment after IORT; WBRT = whole breast radiation therapy.

Patients had to be 40 years of age or older. Mammography, ultrasound, and magnetic resonance imaging (MRI) (unless medically contraindicated) were required for all patients. Tumor extent had to be ≤ 30 mm on all imaging studies and by final histopathology. All invasive cancers required a negative sentinel lymph node by intraoperative frozen section. The IORT balloon-to-skin distance had to be ≥ 8mm in every direction as measured by ultrasound. Final histopathology had to confirm margin width ≥ 2 mm, negative axillary lymph nodes, [N0(i+) isolated tumor cells acceptable], no extensive lymphovascular invasion (LVI), defined as three or more foci, and not multifocal/multicentric. In late 2022, the requirements were tightened, requiring age ≥ 50 years, tumor span ≤ 20mm, luminal A if invasive, grade 1 or 2, no genetic predisposition to breast cancer, and invasive lobular carcinoma was prohibited. Failure to meet any of these criteria was a protocol deviation, triggering a recommendation for additional treatment, depending on the nature of the violation. This could be whole breast radiation therapy (WBRT), re-excision, WBRT plus re-excision, or mastectomy. This resulted in eight different treatment groups (Fig. 1A, Table 1). Figure 1B simplifies this into two groups: early-breast cancers that received local treatment only (bold lettering in bold rectangular boxes) and those who received whole breast treatment (bold lettering in bold ovals). Additional treatment depended on the exact protocol deviation and was decided upon by the treating team and patient. The goal for all patients was a single treatment: excision of the primary tumor, IORT, and no additional local treatment.

**Table 1:** 1828 Tumors: type of local breast treatment and whole breast treatment and number of local recurrences within each group.

Local treatment	*N* = 1527 (83.5%)	No. recurrences
Excision plus IORT	1474	93
Excision plus IORT plus re-excision	45	6
Excision alone, No IORT or additional WBRT	8	1

Whole breast treatment	*N* = 301(16.5%)	No. recurrences
Excision plus IORT plus WBRT	192	4
Excision plus WBRT, No IORT	66	1
Excision Plus IORT Plus re-excision Plus WBRT	18	0
Excision plus IORT plus Mastectomy	23	1
Excision plus Mastectomy, No IORT	2	0

IORT - intraopertive radiation therapy; WBRT - whole breast radiation therapy.

### Procedure

After primary tumor excision, an IORT balloon and chest wall shield were placed as has been previously described,^18^ 20 Gy (50kV) X-ray irradiation was administered using the Xoft Axxent Electronic Brachytherapy System^®^ (Xoft, San Jose, CA, a subsidiary of iCAD, Inc.). Average treatment time was 10 min.

### Endpoints

Recurrence was defined as any invasive or in situ event in any quadrant of the ipsilateral breast. Axillary and distant recurrences, breast cancer deaths, deaths from other causes, and side-effects were recorded.^20^ Kaplan-Meier analyses were used to estimate local recurrence and survival probabilities. Log-rank testing was used to compare curves. The main analysis was done on an intention-to-treat basis and included all patients, regardless of what treatment they did or did not receive. Additional analyses were done by subgroups, for example, tumors that received whole breast treatment versus those that received partial breast treatment, DCIS only, IORT only, ASTRO recommended, etc.

## Results

A total of 1828 breast cancers in 1785 patients (43 bilateral) were treated between June 2010 and May 2024: 1667 patients received IORT during initial lumpectomy, 85 received delayed IORT during a second surgery, and in 76 patients, IORT was not given due to a positive sentinel node on frozen section (*n* = 62), skin distance < 8mm (*n* = 13), or equipment failure (*n* = 1).

All 1828 underwent excision of the primary tumor. The median follow-up was 85 months (7.1 years). 1805 (99%) were followed more than 1 year, and 1293 (71%) more than 5 years. A total of 1481 (81%) tumors were invasive and 347 (19%) were DCIS. Median age was 66 years and median size was 16 mm. In addition, 905 (50%) tumors were ASTRO recommended for accelerated partial breast irradiation (APBI) using the recently updated 2024 guidelines^14^, and 96% were estrogen receptor positive and 86% were progesterone receptor positive. Finally 98% of invasive tumors were luminal cancers, of which 74% were luminal A. Characteristics of the entire cohort are detailed in

Table 2.

**Table 2:** Characteristics of IORT Trial Cohort.

Variable	*N* (%)
*N*	1828
*Tumor type:*	
DCIS	347 (19%)
Infiltrating ductal	1328 (73%)
Infiltrating lobular	153 (8%)
Median follow-up (range)	85 months (4.3 Mo – 14.5 Years)
Median follow-up ≥ 1 year	1805 (99%)
Median follow-up ≥ 5 years	1293 (71%)
Median age (range)	66 years (40-92)
Median tumor span	16 mm
*Hormone receptor status*	
Estrogen receptor positive	1749 (95.7%)
Progesterone receptor positive	1577 (86.3%)
*Immediate versus delayed IORT*	
Immediate	1743 (95.4%)
Delayed	85 (4.6%)
*2024 ASTRO APBI categories*	
Recommended	905 (49.5%)
Conditional yes	433 (23.7%)
Conditional no	143 (7.8%)
Not recommended	347 (19.0%)
*Biologic subtype (invasive only)*	
Luminal A	1093/1481 (73.8%)
Luminal B (HER2 Neg)	316/1481 (21.3%)
Luminal B (HER2 Pos)	38/1481 (2.6%)
HER2 Pos, ER/PR Neg	3/1481 (0.2%)
Triple negative	31/1481 (2.1%)

Multiple different treatments occurred because intraoperative events or final histopathology yielded findings that precluded excision plus IORT as the only local treatment. All patients with protocol deviations were advised to have additional local treatment. This was complicated by patient and physician choice as to what the additional treatment should be, if any. For example, 476 patients had protocol deviations discovered by final histopathology (top central box under title, Fig.1A). In total, 211 of these patients declined any additional local treatment, while the remaining 265 chose 4 different treatment approaches (Fig. 1A).

**1276 Tumors that Met All IORT Protocol Requirements**

A total of 1276 (70%) tumors met all Hoag Protocol requirements and were advised that their local treatment was complete. Despite meeting all IORT requirements, 13 patients elected additional treatment: 8 added WBRT, 2 underwent re-excision, and 3 converted to mastectomy.

**552 Tumors that Did NOT Meet All IORT Protocol Requirements**

The remaining 552 tumors (30%) experienced a total of 690 protocol deviations

(Table 3). 476 received IORT, as the deviation was not discovered until final histopathology (top central box under title, Fig.1A), and 76 experienced a deviation in the operating room and IORT was not given (top right box under title, Fig. 1A). Among 552 patients who deviated from protocol requirements, 219 (40%) refused any additional local breast treatment and 333 accepted additional local treatment, including re-excision (*n* = 43), re-excision plus WBRT (*n* = 18), WBRT (*n* = 250), or mastectomy (*n* = 22) (Figure 1A)

**Table 3:** Protocol deviations and treatment. 690 deviation among 552 patients.

Protocol deviation	690 Deviations
Margins < 2 mm	266
Tumor extent > 30 mm	226
Positive lymph nodes ≥1	118
Extensive lymphovascular invasion ≥3 foci	50
Multifocal/multicentric	13
Skin to balloon distance < 8mm	16
Equipment failure	1

Treatment following protocol deviation	552 Patients
Refused additional local treatment	219 (39.7%)
Accepted additional treatment	333 (60.3%)
Re-excision plus WBRT	18
Re-excision	43
WBRT	250
Mastectomy	22

WBRT = whole breast radiation therapy.

The eight treatment groups cannot be accurately analyzed individually. However, they can be grouped into two different cohorts that can be analyzed. The first group includes 1527 tumors that received local treatment only. The part of the breast with the index tumor was the only part of the breast treated [IORT only (*n* = 1474)], IORT plus re-excision [*n* = 45], and excision alone—(no IORT or WBRT) [*n* = 8] (Table 1A).

The second group includes 301 tumors that received local treatment followed by whole breast treatment. These include those who received IORT followed by WBRT (*n* = 192), those who did not get IORT in the operating room but received WBRT (*n* = 66), those who received IORT plus re-excision and then WBRT (*n* = 18), those who received IORT followed by conversion to mastectomy (*n* = 23), and 2 patients that had IORT cancelled in the operating room and opted for mastectomy (Fig. 1B, Table 1)).

### Recurrence and Survival

**Fig. 2:**
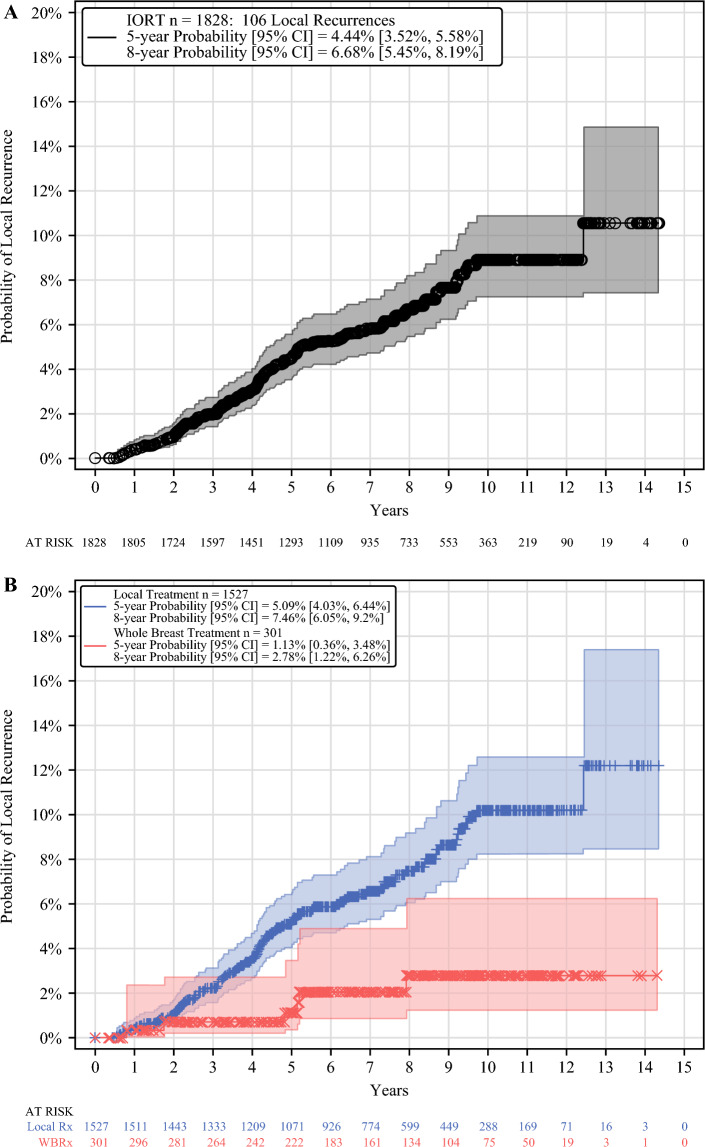
**A** Kaplan–Meier curve for 1828 breast cancers that shows the probability of any local recurrence (Invasive or DCIS) in any quadrant. 5 -year probability of local recurrence was 4.44%; **B** 1828 breast cancers were split into two groups: 1527 tumors (blue curve) that received local treatment to the segment of the breast with the index tumor versus 301 tumors (red curve) that received whole breast treatment; probability of local recurrence at 5-years for those who received local treatment was 5.09% compared with1.13% for those who received whole breast treatment (*p* = 0.001); this difference is notable since the patients receiving whole breast treatment were the most likely to recur, having had poor prognostic findings on final histopathology that initiated the need for whole breast treatment.

There were 106 ipsilateral breast tumor recurrences: 87 (82%) invasive and 19 (18%) DCIS; 77 (73%) recurrences were in the same quadrant as the index cancer while 29 (27%) were in different quadrants. The Kaplan–Meier probability of local recurrence for all 1828 tumors at 5 and 8-years was 4.44% and 6.68%, respectively (Fig. 2A). The tumors were subdivided into two groups (Fig. 2B): 301 who received whole breast treatment and 1527 received local treatment only. The 5 and 8-year probabilities of local recurrence was 1.13% and 2.78% for whole breast treatment and 5.09% and 7.46% for local treatment, respectively (*p*= 0.001). In a previous publication, whole breast treatment was shown to be the single most important predictor of local recurrence by multivariate analysis^17^

The 5 and 8-year probabilities of local recurrence using a variety of subgroupings are reported in Table 4. DCIS recurred at a 5-year rate of 4.71%, slightly higher than the invasive cancer rate of 4.38%, but not significantly different (*p* = 0.95). The subgroup with the lowest rate of recurrence consisted of patients ≥ 65 years with luminal A biology and a tumor span ≤ 20 mm. This group had a 5-year probability of local recurrence of 2.32%. The 5-year local recurrence probability can be even lower at 1.24%, if 10 mm or more final histopathologic margins are required.

**Table 4:** Kaplan-Meier 5-Year and 8-year probability of local or distant recurrence or survival for subgroups.

Category	*N*	No. local recurrences	5-year probability (95% CI)	8-year probability (95% CI)
All local recurrences (DCIS + Invasive) all quadrants	1828	106	4.44% (3.52%-5.58%)	6.68% (5.45%-8.19%)
Whole breast treatment	301	6	1.13% (0.36%-3.48%)	2.78% (1.22%-6.26%%)
Partial breast treatment	1527	100	5.09% (4.03%-6.44%)	7.46% (6.05%-9.20%)

All patients and recurrences by quadrant location and type	*N*	No. local recurrences	5-year probability	8-year probability
All local recurrences (DCIS + Invasive) same quadrant	1828	77	3.11%	4.48%
Invasive local recurrences all quadrants	1828	87	3.43%	5.54%
Invasive local recurrences same quadrant	1828	66	2.56%	4.17%

Pure DCIS patients		No. local recurrences	5-year probability	8-year probability
DCIS tumors (all recurrences)	347	21	4.71%	6.43%
DCIS tumors (invasive recurrences)	347	13	2.54%	3.89%

Subgroups receiving local treatment only (No WBRx)		No. local recurrences	5-YEAR PROBABILITY	5-year probability
IORT only patients	1474	93	5.03%	7.11%
Met Hoag IORT requirements	1265	73	4.47%	6.55%
ASTRO 2023 Recommended	888	44	3.81%	5.50%
ASTRO 2023 Not Recommended	108	14	9.36%	15.79%
Luminal A (Invasive Only)	929	45	3.97%	5.38%
Luminal A (Invasive Only) and ≥ 65 Years	543	19	3.38%	4.04%
Luminal A (Invasive Only) and ≥ 65 Years and Tumor Span ≤ 20mm	407	13	2.81%	3.71%
Luminal A (Invasive Only) and ≥ 65 Years, Tumor Span ≤ 20mm, and 10mm Margins	204	3	1.24%	2.14%

Axillary and distant recurrences	*N*	No. axillary or distant recurrences	5-year probability	8-year probability
Axillary recurrences	1828	14	0.25 %	0.89%
Distant recurrences	1828	16	0.57%	0.97%

Survival	*N*	No. deaths	5-year probability	8-year probability
Breast cancer specific survival	1828	7	99.7%	99.7%
Overall survival	1828	105	97.0%	93.3%

DCIS - ductal carcinoma in situ; Inv – invasive; WBRx - whole breast treatment; IORT - intraoperative radiation therapy; CI – Confidence Interval.

The subgroup recurring at the highest rate was 108 ASTRO “Not recommended for APBI” patients who declined additional whole breast treatment. Their 5-year probability of local recurrence was 9.36%. For 239 ASTRO “Not recommended for APBI” who accepted whole breast treatment, the 5-year local recurrence rate was 0.42% (*p* <0.0001).

At 8-years, the axillary and distant recurrence probabilities were less than 1%, breast cancer specific survival was more than 99%, and overall survival was 93.3% (Table 4).

### Treatment for 106 Patients with Local Recurrences

In total, 33 patients with local recurrence were treated with excision of the local recurrence alone, 31with excision plus WBRT, and 37 with mastectomy. Two patients with serious conditions, unrelated to breast cancer, did not have their recurrences excised. Two patients with simultaneous metastatic breast cancer went directly to chemotherapy. One patient with a low-grade DCIS recurrence in another quadrant was treated with tamoxifen and has not recurred for 10-years. Three of 106 patients with local recurrences have died from breast cancer, all of whom presented with simultaneous local recurrence and distant metastatic disease. Four patients are alive with metastatic breast cancer, 3 of whom presented with local recurrence and metastatic disease simultaneously, and 65% of patients with a local recurrence were treated with repeat breast conservation.

## Discussion

Two prospective randomized trials, TARGIT-A and ELIOT support the concept of IORT and have yielded acceptable long-term recurrence rates^2–4,6,21^. TARGIT-A used 50 kV photons and intention-to-treat methodology in their analysis. They reported the lowest 5-year probability of local recurrence in the literature at 2.11%, but 30% of their patients received additional whole breast radiation therapy.^3^ The addition of whole breast treatment is called ‘individualized risk-adapted IORT’. Whole breast treatment is added when high-risk features are found by final histopathology. This is generally the addition of WBRT but could be conversion to mastectomy. The addition of whole breast treatment lowers the risk of local recurrence but intention-to-treat analysis keeps that patient in the IORT arm of the trial. The higher the percentage of patients receiving whole breast treatment, the lower the overall local recurrence rate. TARGIT-A has been criticized because the percentage of patients receiving additional WBRT was relatively high.^11,12^

The ELIOT trial, which used electrons, reported a 4.2% recurrence probability at 5-years, twice as high as TARGIT-A, but only 5% of their patients received additional WBRT. Their results were acceptable to many and based on these two trials IORT flourished in Europe. When ELIOT reported a 15-year local recurrence of 12.6% for patients treated with IORT compared with 2.4% for WBRT (*p* < 0.0001)), ^4^, support faded in the USA, and this was likely an important reason why ASTRO decided not to recommend IORT in their 2024 Partial Breast Irradiation Practice Guidelines.^14^

In the study reported here, we experienced a local recurrence probability of 4.44% and 6.68% at 5 and 8-years, respectively, for the entire cohort of 1828 breast cancers. When these 1828 tumors are subdivided by those who received local treatment to the area of the primary tumor only versus whole breast treatment, the results are significantly different. For 1527 tumors receiving only local treatment to the area of the primary tumor, the 5-year local recurrence probability was 5.09%. For whole breast treatment, it was 1.13% (*p* = 0.001). This approximately 4–5-fold difference in local recurrence was discussed with our patients, most of whom found it acceptable and offset by the multiple benefits of IORT. The low rate of local recurrence in those who received whole breast treatment (1.13%) is notable, since these were the patients with protocol violations (poor prognostic factors) and those most likely to recur.

As trials of hypofractionation matured,^22–24^ external beam APBI delivered over 5 fractions as done in the Florence trial became the preferred method for external beam partial breast irradiation in the USA. The Florence trial reported a local recurrence rate at 10-years of 3.7% in the APBI arm versus 2.5% for WBRT (*p* = 0.40).^22,25^

When coronavirus disease 2019 (COVID-19) arrived in 2020, IORT became a sought-after treatment at our facility.

A single trip to the operating room for all local treatment, limiting COVID exposure, was very appealing when compared with the alternative of surgery, a period of healing, and then multiple trips to the radiation therapy center.

The choice of IORT versus a more standard radiation therapy approach was never about electing a procedure with a greater risk of dying. The overall survival and breast cancer specific survival in Target-A and ELIOT have always been equivalent^2–4^. Multiple other modern studies reveal that regardless of the type of radiation therapy or surgery done to the breast, survival is equivalent. It is only the rate of local recurrence that varies. Among the 106 local recurrences that we have experienced, only 3 have gone on to die from breast cancer. In all three, distant disease was present at the time local recurrence was diagnosed, making it unlikely that local recurrence was the source of their distant disease.

With IORT, should there be a local recurrence, the recurrence can generally be excised and standard radiation therapy given, with minimal impact on survival. The Breast Cancer Trialists have reported that for every four local recurrences there is one extra death at 15 years.^26^ This is based on data from the last century and may not apply to the highly favorable tumors that we are selecting for IORT today. Assuming the Trialists’ results do apply, if IORT were to be limited to patients 70 years or older, there would be little likelihood of a local recurrence leading to a breast cancer death. Our breast-cancer-specific survival at 8 -years was 99.7% despite 106 local recurrences. This speaks to the safety of IORT and the treatability of a local recurrence in these patients.

We understand the profound impact of local recurrence. When they do occur, they are depressing for the patient and the treating team, cause a feeling of failure, create fear of dying, and require additional treatment.

IORT offers many benefits. It is quick, simple, less expensive, less damaging to surrounding tissue, offers a better quality of life, better cosmesis, especially with breast augmentation, less travel to and from treatment, therefore “greener,” and so on. The downside for the patient is a higher local recurrence rate but with little or no impact on survival. The downside for the surgeon, the radiation oncology team, and the hospital is added time to the procedure with poor reimbursement. Our team considered all of these factors.

Overall, we found IORT to be a valuable tool. As a group, we felt the relatively low risk of local recurrence was acceptable in exchange for the benefits IORT offered. We accepted the extra time that the radiation oncology team had to spend away from the cancer center, the additional surgical time during cavity preparation, the large amounts of time spent in preoperative counseling, the inevitable operating room delays, the difficulties in scheduling, and the low reimbursement rate. Regardless of these factors, we would have been willing to continue the IORT program, but with ASTRO’s 2024 recommendation against IORT, we felt we could no longer struggle against the prevailing trend in this country. As of June, 2024, we no longer offered IORT at our facility.

The study reported here has several limitations. It was a retrospective analysis of a single-arm IRB-approved registry with no ability to compare IORT with other irradiation approaches or excision alone. Patient willingness to accept additional whole breast treatment when suggested varied. Toward the end of the study, entry requirements became stricter.

## Conclusions

IORT is an extremely convenient form of radiation therapy, offering patients many advantages when compared with other methods of partial or whole breast treatment. Some of IORT’s benefits have been offset with the development of new shorter hypofractionation regimens. In this series, the 5-year local recurrence rate was 4.44%, about four times higher than what would be expected with standard forms of radiation therapy. Despite this, breast-cancer-specific survival was 99.7% at 8-years. In 2024, because of the higher local recurrence rate, ASTRO decided not to recommend IORT outside of a clinical trial. Without ASTRO’s support, our group felt that we could no longer continue offering IORT.
